# PFGPred: a stack ensemble classifier for the identification of fusion genes in plants

**DOI:** 10.1093/dnares/dsag005

**Published:** 2026-06-09

**Authors:** Fiza Hamid, Kanka Mukherjee, Sakshi Chaudhary, Love Kaushik, Shailesh Kumar

**Affiliations:** Bioinformatics Lab, BRIC-National Institute of Plant Genome Research, Aruna Asaf Ali Marg, New Delhi 110067, India; Bioinformatics Lab, BRIC-National Institute of Plant Genome Research, Aruna Asaf Ali Marg, New Delhi 110067, India; Bioinformatics Lab, BRIC-National Institute of Plant Genome Research, Aruna Asaf Ali Marg, New Delhi 110067, India; Bioinformatics Lab, BRIC-National Institute of Plant Genome Research, Aruna Asaf Ali Marg, New Delhi 110067, India; Bioinformatics Lab, BRIC-National Institute of Plant Genome Research, Aruna Asaf Ali Marg, New Delhi 110067, India

**Keywords:** fusion transcripts, gene fusion, machine learning, plant fusion gene, whole-genome sequencing

## Abstract

Fusion genes play crucial roles in plant biological processes but remain far less explored than their human counterparts, largely due to limited validated datasets and the absence of plant-specific prediction tools. Existing approaches often produce high false-positive rates, restricting reliable discovery. To address this gap, we developed *Plant Fusion Gene Predictor (PFGPred).* This ensemble machine learning framework integrates Random Forest, XGBoost, and long short-term memory (LSTM) models into a meta-classifier for accurate identification of true and false fusion genes from RNA sequencing (RNA-Seq) data. PFGPred was trained on a high-confidence dataset of fusion genes validated by both RNA-Seq and whole-genome sequencing from *Arabidopsis thaliana*, *Oryza sativa*, *Triticum aestivum*, and *Zea mays*, to predict and rank candidate fusion genes for future functional validation. It outperformed individual baseline models, achieving accuracies of 0.97 on training data and 0.77 on independent test data. When evaluated on human datasets, it achieved 0.71 accuracy at the cost of lower sensitivity, reflecting biological differences between plant and human fusion events. Comparative analyses confirmed that PFGPred reliably identifies validated fusions, demonstrating its utility as a cost-effective, plant-specific prediction tool for high-throughput fusion gene screening and functional genomics research. It is freely available as a web server at http://www.nipgr.ac.in/PFGPred.

Key Points

- In plants, the identification of fusion genes is challenging due to the high rate of false positives by existing computational methods and the lack of plant-specific prediction tools.
- Here, we proposed PFGPred, a novel computational pipeline to improve generalization in plant fusion identification. It integrates machine learning and deep learning classifiers trained on fusion-related features, including junction, expression, genomic, and structural features derived from fusion genes across four plant species. The model is trained and tested on a mixed dataset capturing both intra- and inter-species fusion patterns across multiple plant species.
- PFGPred outperformed its constituent baseline models on both training and independent test datasets and showed an accuracy of 0.97 and 0.77, respectively. PFGPred correctly identified most validated fusions, underscoring its value as a cost-effective tool for fusion gene prediction using only RNA sequencing data.
- PFGPred will facilitate high-throughput screening of true fusion genes and advance research on the functional characterization of their encoded products. This pipeline is freely available on GitHub (https://github.com/skbinfo/PFGPred). It is provided as a user-friendly web server at http://www.nipgr.ac.in/PFGPred.

## 1. Introduction

Several molecular mechanisms are known for the evolution of new genes, including gene duplication and divergence, de novo evolution, and recombination events such as gene fusion and gene fission.^1–4^ New genes formed by combining sequences from two separate genes are called fusion genes or chimeric genes. They may encode a fusion protein with a unique domain architecture or act as regulatory RNA molecules.^5,6^ Previously seen as artifacts or evolutionary anomalies, fusion genes are now recognized across all domains of life, from bacteria^7^ to plants^8^ and animals.^9^ For decades, researchers focused on the oncogenic aspects of fusion genes in humans.^5^ With advances in high-throughput sequencing, it is now evident that fusion events are far more common in plants than previously recognized and contribute to genome innovation and trait evolution.

Genome-wide investigation across multiple rice genomes revealed a high rate of fusion gene origination, estimated at 63 fusion genes per million years.^3^ In *Oryza sativa*, 50% of newly formed genes on the short arm of chromosome 3 have chimeric gene structures with the potential to acquire novel functions.^10^ Pan-genome and transcriptome studies across multiple plants showed a high level of diversity in chimeric RNA profiles within a species. Intra-species clustering of accessions based on fusion events closely matched clustering from genomic variants, highlighting a link between fusion events and genetic variations.^11,12^ Functional studies of fusion genes have shown their diverse roles in plants, influencing development and growth,^11,13–15^ immunity,^16,17^ stress adaptation,^3,11,18^ and metabolic regulation.^8^ Despite growing evidence of their relevance, fusion genes in plants have received limited attention. Hence, extensive experimental validation is required to establish their biological significance. Because large-scale experimental validation is resource-intensive, computational approaches provide an efficient alternative for identifying high-confidence fusion genes from multi-omics datasets.

Currently, many fusion detection tools are available, but only EricScript-Plant is specifically designed for plants.^19,20^ These tools largely rely on high-throughput datasets, including RNA sequencing (RNA-Seq) and whole-genome sequencing (WGS).^21^ Tools that use RNA-Seq data offer the advantage over WGS-based tools by enabling the identification of expressed fusion transcripts and quantifying their expression. However, they are prone to false-positive detections arising from read misalignment due to repetitive sequences, paralogous genes, or overlapping genes, as well as from incomplete genome annotation and sequencing artefacts resulting from template switching during library preparation. Also, it cannot resolve genomic breakpoints located within introns. Conversely, WGS allows precise identification of genomic breakpoints but cannot distinguish between expressed and non-expressed events and frequently captures non-functional genomic rearrangements. Because WGS and RNA-Seq each have inherent limitations when used independently, integrating both datasets for orthogonal validation can improve detection accuracy, as fusion transcripts supported by corresponding genomic breakpoints provide strong evidence of true fusions.^22^ Since the integrative approach is expensive and computationally demanding, we developed a machine-learning classifier trained on features from fusion genes validated by both datasets, allowing accurate prediction using only RNA-Seq data. Conventional fusion detection tools generate many false positives and produce very different predictions when applied to the same datasets. Our method can be applied to the output of any standard RNA-Seq-based fusion detection tool and helps researchers prioritize high-confidence candidates.

Machine learning (ML) approaches have become popular in addressing complex biological problems, including the prediction of lncRNAs, open reading frames, and peptides in plants.^23–26^ Despite the robustness of building ML models that are both accurate and generalizable, they are not yet implemented for plant fusion gene identification. Inspired by recent advances demonstrating the use of ML for accurate fusion detection in cancer,^27,28^ we constructed an ensemble-based model that generalizes across diverse plant species to robustly identify fusion genes. By integrating multiple base models into a single meta-model to leverage their complementary strengths, the ensemble approach improves overall predictive performance. Here, we developed PFGPred, a plant fusion gene prediction framework based on a stacked ensemble of XGBoost, Random Forest (RF), and long short-term memory (LSTM) models. We compared the predictive accuracy of the meta-model with that of its constituent base models to evaluate the performance gains achieved through stacking. To evaluate the model's generalizability, we tested its performance across multiple plant species and human datasets. We also compared the performance of PFGPred with fusion gene prediction tools that require both WGS and RNA-Seq data. Using RNA-Seq alone, the model correctly identified most fusion events validated by integrative methods. Providing a probability score for each prediction enables researchers to focus on accurate fusion genes for downstream functional studies.

## 2. Materials and methods

### 2.1. Framework design

**Fig. 1.**
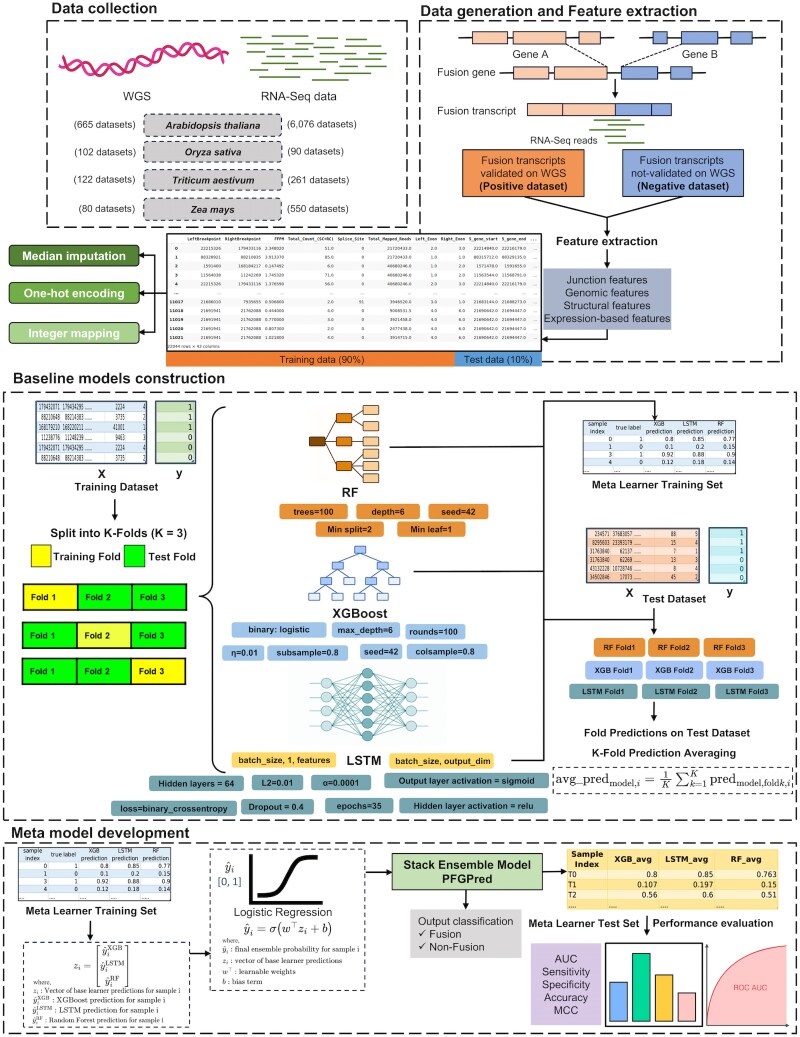
An overview of the PFGPred stacking ensemble framework for predicting fusion genes in plants. The workflow includes data collection and preprocessing, data construction, feature extraction, baseline and meta-model development, and performance evaluation.

PFGPred is an ML framework for detecting fusion genes using RNA-Seq data. The PFGPred pipeline includes several steps: data collection and preprocessing, generating positive and negative datasets, feature extraction, model development, and performance evaluation. First, fusion transcripts were identified from RNA-Seq data and then validated using WGS data to separate true fusion events from false positives. For both WGS-validated and non-validated fusions, a comprehensive set of features was derived from RNA-Seq data and genome annotation. The predictive model was trained on this dataset using an ensemble-based approach that integrates RF, XGBoost, and LSTM networks to enhance prediction robustness. The performance of PFGPred was evaluated on independent datasets using standard evaluation metrics, including sensitivity, specificity, accuracy, Matthews correlation coefficient (MCC), and area under the curve (AUC) (Fig. 1).

### 2.2. Data collection and processing

WGS and RNA-Seq data from multiple accessions of *Arabidopsis thaliana*, *O. sativa*, *Zea mays*, *Triticum aestivum*, *Cicer arietinum*, *Glycine max*, and *Setaria italica* were retrieved from the NCBI Sequence Read Archive. For *A. thaliana*, data from the 1,001 Genomes Project were used for model training, while for *O. sativa*, data from 33 accessions included in the rice pan-genome study were used. For *Z. mays*, *T. aestivum*, *C. arietinum*, and *G. max*, datasets from multiple independent studies were used. For *S. italica*, data were obtained from BioProject PRJNA633413 and PRJNA633940. Separate sets of WGS and RNA-Seq data were used for model training and independent testing to ensure unbiased evaluation, as listed in Table S1.

The data were downloaded in FASTQ format, quality-checked, and trimmed using FastQC (v0.11.9) and TrimGalore (v0.6.10). RNA-Seq datasets were analysed for fusion transcript detection using FusionMap (OShell version 12.3.0.13)^29^ and STAR-Fusion (v1.12.0).^30^ To validate these fusion transcripts at the DNA level, WGS data from each accession were used for fusion gene validation. Initially, the WGS data were aligned to their respective reference genomes using BWA-MEM (v0.7.17),^31^ followed by fusion gene detection using the WGS validation pipeline.^22^ The validated fusion gene pairs were further filtered to eliminate likely false positives, including those arising from paralogous genes and overlapping genomic regions. Read-through candidates were retained during initial screening, and WGS support served as the primary criterion for distinguishing high-confidence genomic fusion events from likely transcriptional artifacts.

### 2.3. Construction of training and test datasets

For each species, fusion events confirmed by both RNA-Seq and WGS data were considered as true positives (positive dataset), and those identified in RNA-Seq only were treated as false positives (negative dataset) for model construction. As the negative dataset (non-validated fusions) was larger than the positive dataset (validated fusions), stratified sampling was used to create a balanced dataset containing equal numbers of positive and negative samples for model training. The final dataset was split into 90% for model construction (the training dataset) and 10% for performance evaluation (the test dataset).

To assess the model's cross-species performance, independent datasets from multiple plant species were used, including *C. arietinum*, *G. max*, and *S. italica*. Additionally, to evaluate the generalizability of our stacked ensemble framework for human fusion gene detection, a separate model was trained using the human dataset described by Kim et al.^27^ RNA-Seq and WGS data from multiple human cancer cell lines were aligned to the GRCh38.p14 reference genome using BWA (v0.7.17).^31^ Performance of the human-specific model was further validated using an independent dataset reported by Hafstað et al.^22^ All datasets used in this study are available in the GitHub repository.

### 2.4. Feature extraction

To extract informative features that can effectively distinguish true fusions from false positives, we used features derived from fusion detection tools and associated genome annotations. In total, 27 features were taken and grouped into four categories: (i) junction features, (ii) genomic features, (iii) structural features, and (iv) expression-based features (Table 1). After feature extraction, the data were preprocessed before passing through the model (detailed in Method S1). After the preprocessing steps, the total number of input features increased to 41. Further, importance scores were calculated for each feature using the mutual information criterion (Method S2). The complete list of features, along with their importance score, is provided in Table S2. The model was then trained and evaluated on an independent test dataset using the top 10, 20, 30, and all features ranked by their importance scores, allowing assessment of the model's performance with varying feature sets.

**Table 1.** A detailed description of all the fusion-related features used in model construction.

Category	Description	Feature
Junction features	Precise location of the fusion breakpoint in the genome, the splice site sequence at the junction, and the number of fusion isoforms identified for each gene pair.	Left breakpoint Right breakpoint Splice site • Alternate junction count
Genomic features	Positional and structural relation between the partner genes, including whether the fusion event was inter- or intra-chromosomal, the genomic distance separating the partners (for intra-chromosomal fusions), and the strand orientation (sense or antisense) of each gene. Includes parental gene details such as genomic coordinates, gene length, and exon number.	Chromosomal feature (inter- or intra-chromosomal) Same strand 5′ gene start, end, length, exon count 3′ gene start, end, length, exon count
Structural features	Exonic location of the breakpoint (exon number of breakpoint, exon boundaries), the reading frame status of the fusion (in-frame or out-of-frame), and the splice pattern at the junction (either canonical or non-canonical).	5′ gene exon location 3′ gene exon location Splice pattern (in-frame/out-of-frame) Splice pattern class (canonical/non-canonical)
Expression-based features	Expression-related features, including the number of split reads and spanning read count supporting the fusion junction, and normalized expression level (FFPM).	Total read count FFPM

### 2.5. Model construction

Ensemble models have been reported to demonstrate superior predictive performance compared to individual ML or deep learning (DL) models, as reported in several studies.^23,25^ To leverage this advantage, we implemented a stacking ensemble strategy in which predictions from multiple base learners are combined to generate a more robust final prediction. Heterogeneous ensembles are more robust compared to homogenous ensembles because they capture a wider variety of decision boundaries. Therefore, PFGPred integrates three base models chosen to capture complementary decision patterns: XGBoost for gradient boosted decision tree learning, RF for variance reduction through bagging, and LSTM for capturing nonlinear feature interactions. A comparison of these baseline models and the stack ensemble model with other commonly used models was performed (Table S3). The detailed description of the ensemble model is provided in the supplementary Method S3. Out-of-fold predictions from the base learners were generated using a 3-fold stratified cross-validation to avoid data leakage and provide unbiased meta-training data. The base model predictions were then used as input features for a logistic regression meta-learner, which learned an optimal combination of base model outputs to produce a consensus prediction. The workflow includes four major stages: (i) dataset splitting and feature scaling, (ii) training of base models, (iii) generation of out-of-fold predictions for stacking, and (iv) training of the meta-learner on the stacked predictions to yield the final ensemble output.

In total, three models were constructed for fusion gene identification, including a generalized model applicable across all plant species, a species-specific model for *A. thaliana*, and a human-specific model. For each model, three base models were trained, including XGBoost, RF, and LSTM networks, and their outputs were subsequently integrated using a stacked ensemble learning framework to calculate the final prediction score. The performance of the model was evaluated based on accuracy, precision, sensitivity, specificity, MCC, and AUC. The calculation formulas are provided in Method S4.

### 2.6. Deployment of the stacked ensemble model as fusion detection pipeline

PFGPred was trained on fusion transcripts detected in WGS as well as RNA-Seq data, but only uses features derived from RNA-Seq data and available genomic annotations. This approach ensures the model's applicability for samples lacking WGS data. A custom Python script is provided for fusion transcript identification and feature extraction from RNA-Seq data, with STAR-Fusion integrated as the default detection tool.^32^ To make it a useful addition to conventional software for the identification of fusion detection, the pipeline can process results from any existing fusion detection method. To facilitate the identification of high-confidence fusion genes, PFGPred is available as a web server that incorporates the trained model. Users can also retrain the model on species-specific datasets to enhance prediction accuracy. Together, the integrated pipeline and web server provide a user-friendly platform for reliable, high-confidence fusion gene prediction (Fig. S1).

## 3. Results and discussion

### 3.1. Fusion transcript validation in WGS datasets

For fusion gene detection, we utilized 6,967 RNA-Seq and 969 WGS data derived from diverse accessions of *A. thaliana*, *O. sativa*, *T. aestivum*, and *Z. mays*. First, we employed FusionMap and STAR-Fusion for fusion transcript detection using RNA-Seq data, which resulted in the identification of 45,610 unique fusion transcripts, with species-specific distributions of 13,267 in *A. thaliana*, 5,999 in *O. sativa*, 1,079 in *T. aestivum*, and 25,265 in *Z. mays* (Table 2). These transcripts corresponded to 35,472 distinct gene pairs, comprising 10,583 in *A. thaliana*, 4,749 in *O. sativa*, 869 in *T. aestivum*, and 19,271 in *Z. mays*. Subsequently, the WGS validation pipeline^22^ was employed to verify fusion transcripts at the DNA level using WGS data. This approach enabled the identification of fusion events at the genomic level using WGS data from different accessions. After filtering, a total of 467 fusion genes were validated at the DNA level, including 227 in *A. thaliana*, 50 in *O. sativa*, 37 in *T. aestivum*, and 153 in *Z. mays*. Among these validated fusions, approximately 10% arising from adjacent genes (read-through-like events). The fusion transcript validation rate at the DNA level was strikingly low, which might be due to the high rate of false-positive detection at the RNA level. We further analysed publicly available long-read RNA-Seq data using CTAT-LR-fusion^33^ to cross-validate these DNA-level fusions (Table S4). Moreover, their coding potential was assessed using RNASamba,^34^ which revealed that 84% of these fusion transcripts exhibited high coding scores (>0.90) (Table S5). This suggests that these high-confidence fusions are not merely transcriptional noise, but may contribute to the functional proteomic complexity of plants.

**Table 2.** Summary of the number of RNA-Seq samples analysed, total predicted fusion transcripts, and the subset validated through WGS data.

Plant	Total samples of RNA-Seq	Total fusion transcripts detected in RNA-Seq	Unique Fusion transcripts	Total samples of WGS	Total fusion genes validated in WGS
*Arabidopsis thaliana*	6076	93,334	13,267	665	8,335
*Oryza sativa*	89	14,827	5,999	102	445
*Triticum aestivum*	261	9,808	1,079	122	1,227
*Zea mays*	550	118,676	25,265	80	4,208

### 3.2. Characteristic features of fusion genes

**Fig. 2.**
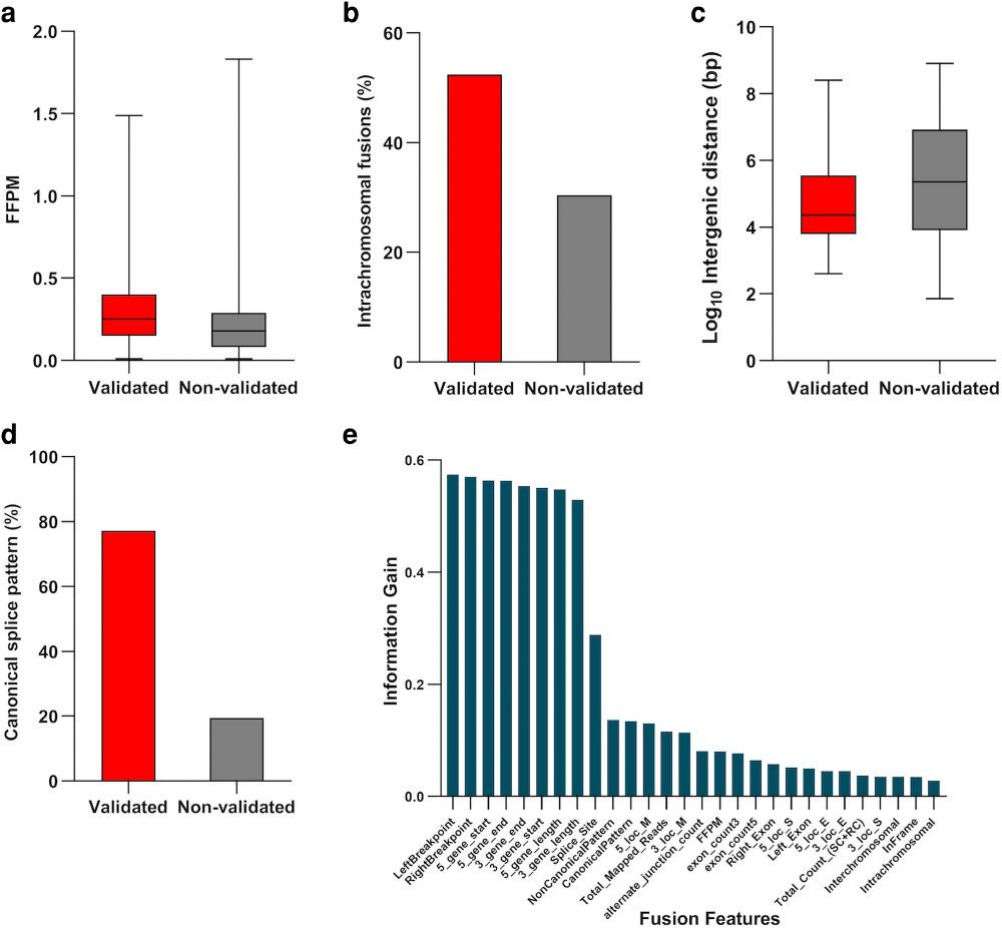
Characteristic features of fusion genes. a) Validated fusion transcripts exhibit higher expression levels (FFPM) compared to non-validated fusions. b) A large proportion of validated fusions are intrachromosomal. c) Genes involved in intrachromosomal validated fusions are closely located in the genome as compared to those of non-validated ones. d) A higher fraction of validated fusions displayed canonical splice patterns at the fusion junction. e) Importance scores of individual fusion-related features based on information gain.

To improve the accuracy of fusion gene identification, we then compared the features of WGS-validated and non-validated fusion transcripts. It was observed that validated fusions showed higher expression in terms of FFPM (fusion fragments per million) than non-validated fusions (*P* < 0.0001, Student's *t*-test) (Fig. 2a). Intrachromosomal fusion events were found to be more common in validated fusion as compared to non-validated events (*P* < 2.2 × 10−16, *χ*^2^ test) (Fig. 2b). Also, intrachromosomal validated fusions tend to occur between genes that are closer together (shorter intergenic distances) (*P* < 0.0001, Student's *t*-test) than non-validated ones (Fig. 2c). Fusion junction analysis revealed that canonical splicing patterns are more abundant in validated fusions (77%) than in non-validated fusions (*P* < 2.2 × 10−16, *χ*^2^ test) (Fig. 2d). Based on these observed differences, we concluded that no single feature could clearly distinguish validated fusion transcripts from non-validated ones. However, a combination of multiple features may capture distinct characteristics that can be leveraged to train an ML classifier for accurately predicting true and false fusion events. To develop the ML classifiers, we extracted a comprehensive set of features for fusion transcripts from RNA-Seq data and associated genomic annotations (Fig. 2e).

### 3.3. A stacked ensemble model for fusion gene detection

**Fig. 3.**
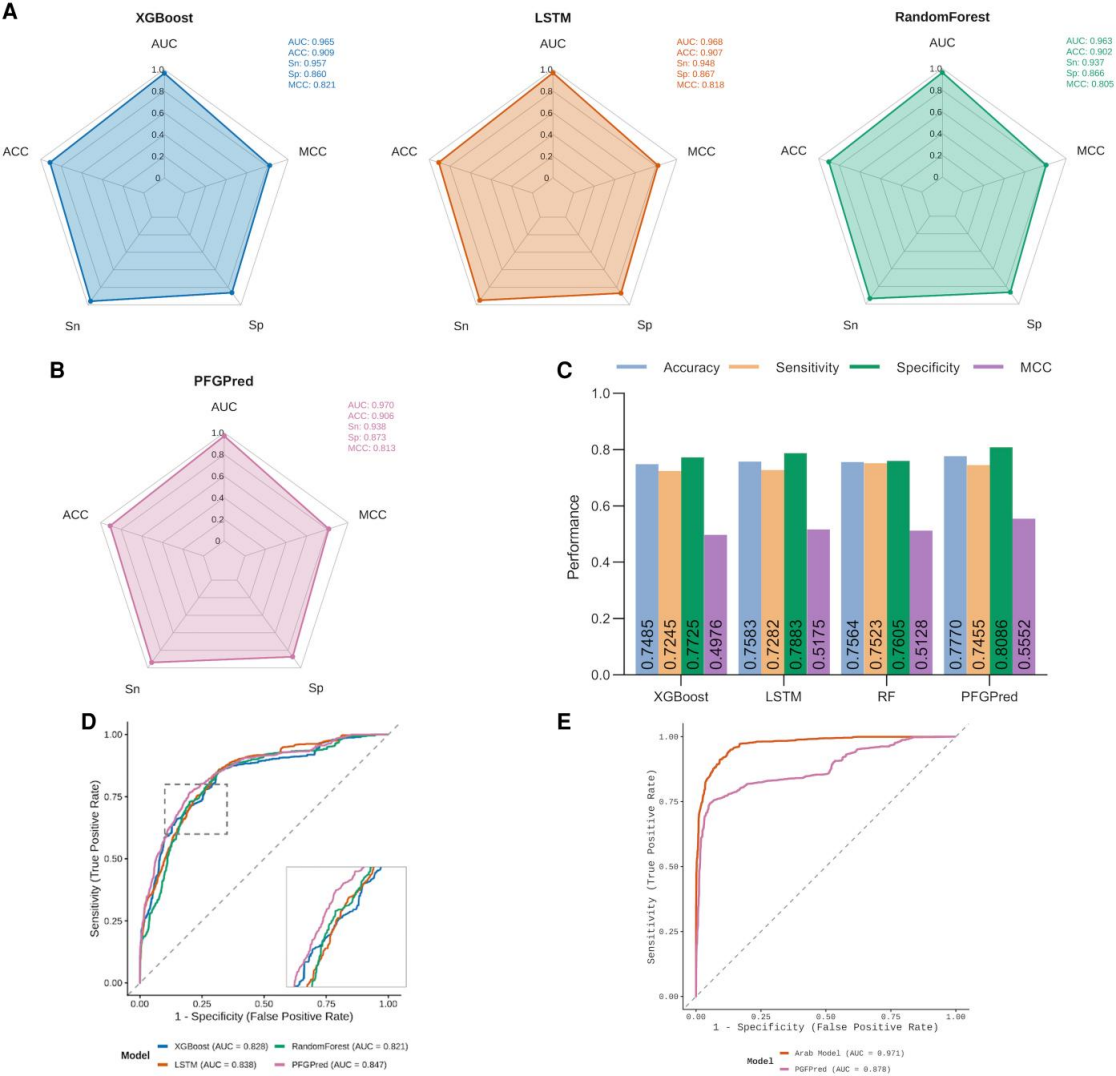
Performance evaluation of the individual base models and stack ensemble classifier (containing XGBoost, Random Forest, and LSTM) for fusion gene prediction in terms of AUC, ACC, Sn, Sp, and MCC on the training dataset a and b) and independent test dataset c and d). e) Performance comparison of the PFGPred model trained on mixed datasets derived from four different plants with the *Arabidopsis*-specific ensemble model.

Due to the absence of experimentally validated fusion genes in plants, the WGS-supported fusion status was considered as the reference ground truth for model training and evaluation. Multiple ML models, including XGBoost, RF, and LSTM networks, were trained on a curated dataset of ∼22,000 fusion events identified across multiple accessions from four plant species. Using the complete set of extracted features as input, the model performance was assessed based on accuracy, sensitivity, specificity, and MCC. Each classifier achieved an accuracy greater than 0.90 during training (Fig. 3a), with RF and XGBoost exhibiting mild overfitting, which was mitigated by tuning tree depth and other hyperparameters.

Rather than selecting a single best-performing model, we adopted a stacked ensemble learning strategy that integrated predictions from multiple base classifiers. This approach leveraged the complementary strengths of ML and DL models, yielding a meta-model with superior predictive power. The stacked ensemble achieved an AUC of 0.97 during training, demonstrating improved performance compared to the individual base classifiers (Fig. 3b). Model performance was further evaluated on independent test datasets. The stacked ensemble outperformed its constituent base models, achieving an accuracy of 0.7762, a specificity of 0.8183, a sensitivity of 0.7342, and an MCC of 0.5545. Among the base models, the LSTM slightly outperformed RF and XGBoost in terms of sensitivity and specificity (Fig. 3c). The Receiver Operating Characteristic (ROC) curve of these models showed AUC increased by over 0.009 to 0.847 after the model ensemble (Fig. 3d).

Furthermore, we compared the performance of each model with different sets of features as input using the top 10, 20, and 30 features ranked by importance score, as well as the complete feature set (Table S6). XGBoost showed slightly better performance with the top 30 features, whereas RF exhibited nearly the same accuracy across all subsets. Both LSTM and the stacked ensemble model achieved the highest accuracy when trained with the full feature set, indicating that diverse feature categories collectively provide critical predictive information (Fig. S2a).

Notably, this model is trained and tested on a mixed dataset capturing both intra- and inter-species fusion patterns across multiple plant species. To further evaluate species-specific performance, we additionally trained the stack ensemble model exclusively on the *A. thaliana* dataset. When evaluated on independent test data, the *Arabidopsis*-trained model showed an increase in the accuracy by 9.29% as compared to our PFGPred model, demonstrating that species-specific training can further enhance prediction accuracy, sensitivity, and specificity (Figs. 3e and  S2b). This suggests that users with sufficient species-specific datasets may retrain the model to capture unique genomic and transcriptomic features of their organism of interest. Detailed instructions for model retraining are provided in the associated GitHub repository.

Further, we evaluated whether cross-species model performance follows evolutionary relationships. Species were grouped into three phylogenetic categories: *A. thaliana* (model eudicot), legumes (*C. arietinum* and *G. max*; eudicots), and grasses (*O. sativa*, *T. aestivum*, *Z. mays*, and *S. italica*; monocots). Standardized equal-sized datasets were generated for each group to control for class-size effects (*n* = 1,964 per group, 982 positive and 982 negative instances). The pre-trained *A. thaliana* model was subsequently evaluated on independent datasets from legumes and grasses. The *Arabidopsis* model achieved higher performance on legumes (AUC = 0.6372) than on grasses (AUC = 0.614). This trend is biologically consistent, as *Arabidopsis* and legumes both belong to the eudicot clade and therefore share a more recent common ancestor than either does with monocot grasses. When the same model was applied to the human dataset, performance declined markedly (AUC = 0.4087). This suggests that the model learned conserved plant fusion characteristics rather than merely memorizing dataset-specific patterns.

Additionally, we assessed whether the relative importance of predictive features is conserved among plants. Using standardized equal-sized datasets, we calculated Pearson correlations of MI scores for the top 20 informative features across plant species and compared them with a human fusion dataset used as an external negative control (Fig. S3). The plant species displayed strong concordance in feature-importance structure, with most inter-plant correlations exceeding 0.85. In contrast, correlations between plant species and the human dataset were consistently lower, declining to 0.682. These results indicate that although certain generic fusion-detection signals may be shared across eukaryotes, the predictive feature architecture of plant fusion events is substantially more similar within plants than between plants and humans.

### 3.4. PFGPred is generalizable and robust when predicting fusions in other organisms

**Fig. 4.**
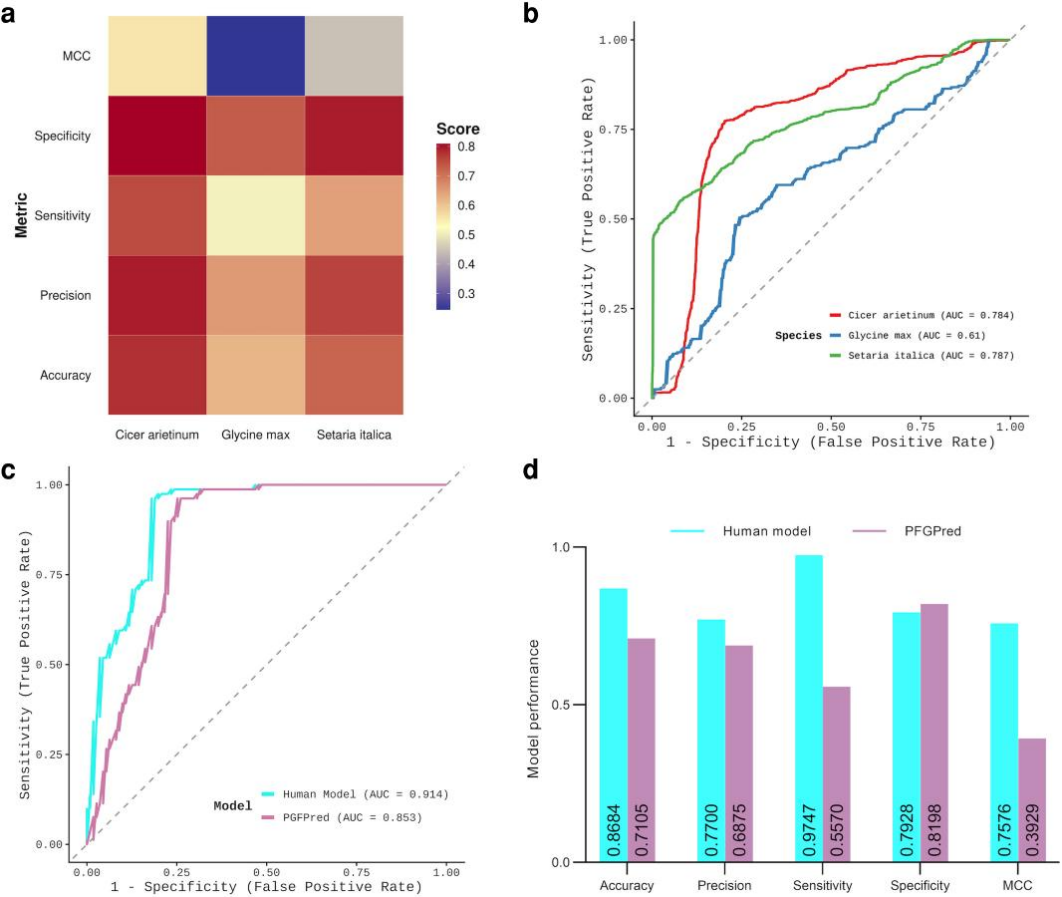
Performance evaluation of PFGPred on the independent datasets. a) The heatmap demonstrates the classification performance of PFGPred on plant species datasets not included during training in terms of accuracy, precision, sensitivity, specificity, and MCC. b) ROC curves and AUC values of the PFGPred for *C. arietinum*, *G. max*, and *S. italica*. c and (d) Performance comparison of PFGPred with a human-specific ensemble model on the human dataset in terms of AUC, accuracy, precision, sensitivity, specificity, and MCC.

To evaluate the generalizability of PFGPred constructed on the *A. thaliana*, *O. sativa*, *T. aestivum*, and *Z. mays* datasets to other species, we tested its performance in other plant datasets, including *C. arietinum*, *G. max*, and *S. italica.* Model performance was evaluated across a range of decision cutoffs to determine the optimal threshold for accurate prediction (Fig. S4). As shown in Figs. 4a and 4b, PFGPred achieved satisfactory prediction performance on the other species, regardless of the evolutionary distance between species, suggesting that PFGPred may be a generalizable computational platform to predict fusion genes in diverse plant species.

We further assessed the cross-species applicability of PFGPred by evaluating its performance on a human dataset and comparing it with a stacked ensemble model trained exclusively on human fusion gene data. With the human dataset, PFGPred showed an accuracy of 0.7105 in predicting fusion genes. In contrast, the human-specific stacked ensemble model reached an accuracy of 0.8684, representing a 15.8% increase (Fig. 4c). This performance gap highlights potential differences in fusion gene patterns between plant and human systems. Interestingly, while the specificity of both models was comparable, PFGPred exhibited markedly low sensitivity on the human dataset. Nevertheless, the ROC curve revealed an AUC of 0.8532, indicating that despite its poor sensitivity, PFGPred still retains considerable predictive utility across species (Fig. 4d).

### 3.5. Comparison of PFGPred with existing methods

We evaluated the performance of our model by comparing it with existing tools that rely on both WGS and RNA-Seq data for fusion gene detection. To assess the overlap of prediction with other existing methods, we used NAFuse^35^ and the WGS validation pipeline,^22^ both of which use a mapping-based approach for fusion identification. By comparing PFGPred against NAFuse and the WGS-validation pipeline, we showed that a machine-learning approach can serve as a resource-efficient alternative for fusion gene detection.

**Fig. 5.**
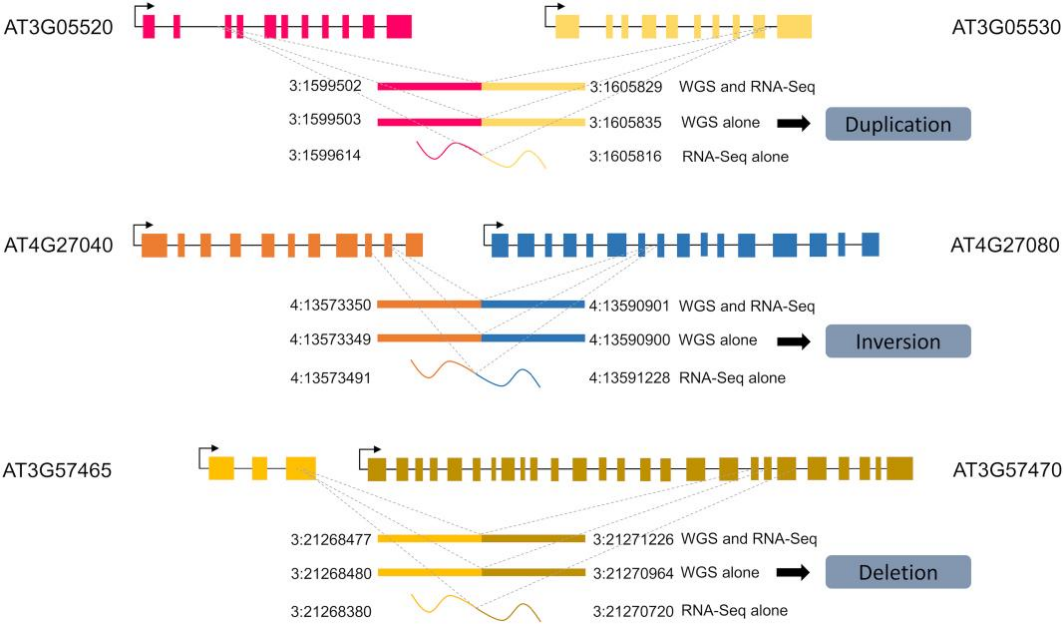
Genomic breakpoints reveal the mechanisms that create fusion genes. Shown are examples of fusion junctions detected using different sequencing strategies, including combined WGS and RNA-Seq, as well as WGS-only and RNA-Seq-only.

At a prediction probability threshold greater than 0.90, our model accurately classified the majority of fusion genes validated by either tool. Notably, all fusions validated by NAFuse (19 fusion genes) were correctly predicted by our model. Among the 42 fusion gene pairs confirmed by the WGS validation pipeline, 37 were accurately classified as true fusions by our model. Furthermore, except for one fusion, all NAFuse-validated fusions overlapped with those identified by the WGS validation pipeline (Fig. S5a). Further, we compared the results of our model to Delly, which is widely used for structural variation identification, to explore the mechanisms associated with fusion genes (Fig. 5). These results showed that our model can successfully detect most fusion genes detected by current methods. Therefore, our approach clearly provides benefit in fusion gene detection without requiring WGS data.

Publicly available plant fusion databases, such as AtFusionDB^36^ and PFusionDB,^37^ offer extensive collections of predicted fusion transcripts based on RNA-Seq data. Currently, AtFusionDB lists about 71,920 unique fusion transcripts in *A. thaliana*, while PFusionDB has around 241,108 fusion transcripts found across various plant species. However, because of the limitations of mapping-based algorithms, many of these fusions may be artefacts or low-confidence events. This presents a major challenge in identifying biologically meaningful candidates for subsequent experimental validation. To evaluate the effectiveness of our stacked ensemble model in supporting existing databases, we analysed the fusion transcripts listed in PFusionDB. The analysis showed that 24.8%, 12.3%, 24%, and 28.4% of the fusion transcripts from *A. thaliana*, *C. arietinum*, *O. sativa japonica*, and *O. sativa indica*, respectively, had a prediction probability score greater than 0.90 (Fig. S5b). A detailed description of the prediction results, including the prediction scores, is in Table S7. This filtering significantly reduces the candidate search space and helps prioritize high-confidence fusions. While PFGPred offers a useful method for discovering plant fusion genes, experimental validation is still crucial for confirming these predictions and revealing their biological significance. Molecular biology techniques such as PCR, Sanger sequencing, and FISH can be exploited for verifying identified fusion genes. A recent study by Cong et al.^11^ experimentally validated several fusion genes in rice through genomic PCR, including *Os11g0106200-Os12g0105800* fusion formed by the translocation of a segment of *Os12g0105800* into the coding region of *Os11g0106200*. In our dataset of diverse rice accessions, this fusion was detected in 30 accessions, including Basmati, N22, TM, and Tumba. Other experimentally validated fusions, such as *BGIOSGA017770-BGIOSGA004569* and *Os08g0534200-Os02g0305800*,^38^ which are associated with abiotic stress responses in rice, were detected by PFGPred with high probability scores (>0.9) (Table S8). These findings demonstrate that PFGPred is capable of prioritizing candidate fusions with potential agronomic and functional significance. Such concordance between computational predictions and independent experimental evidence demonstrates the value of PFGPred in guiding targeted validation.

## 4. Conclusion

Fusion genes arise from genomic rearrangements that juxtapose segments of two separate genes, often resulting in novel transcripts with potential functional implications. In plants, fusion genes are increasingly recognized for their roles in evolution, stress adaptation, and agronomic traits, yet they remain far less explored as compared to animals. High false-positive rates, lack of integration with genomic data, and the absence of plant-specific optimization limit conventional fusion detection tools. Moreover, experimental validation is time-consuming and expensive, making large-scale studies difficult. Therefore, there is a critical need for a computational method that can accurately identify plant fusion genes with minimal false positives.

To address this gap, we developed PFGPred, a plant-specific stacking ensemble model that integrates Random Forest, XGBoost, and LSTM models to accurately distinguish true fusion genes using RNA-Seq data or outputs from existing fusion detection tools. A key challenge in building an ML model was the lack of an experimentally validated plant fusion gene dataset. To overcome this limitation, we leveraged publicly available high-throughput RNA-Seq and WGS datasets from multiple accessions across diverse plant species. By integrating fusion predictions from RNA-Seq with corresponding genomic evidence from WGS, we systematically generated high-confidence positive datasets (true fusions) and curated negative datasets (false predictions). PFGPred was trained on biologically informative features that capture patterns distinguishing true and false fusions across multiple plant species. We conducted a comparative analysis of PFGPred with its constituent baseline models and observed that the stacking strategy improved model performance. Further cross-validation on human and plant datasets highlighted the generalization ability of the model. Notably, PFGPred identified the majority of fusion genes validated by existing integrative methods that require both RNA-Seq and WGS, underscoring its utility as a cost-effective alternative for large-scale fusion discovery. The model architecture can be customized and extended to other species for the identification of novel fusion genes, providing useful insights into gene evolution driven by fusion events. To maximize the utility of PFGPred, the model is provided as a user-friendly web server and has been integrated into a fusion detection pipeline. Taken together, we anticipate that PFGPred will serve as an instrumental bioinformatic toolkit for high-throughput prediction and reliable prioritization of fusion genes, facilitating experimental validation and advancing our understanding of fusion gene biology in plants.

### 4.1. Limitations and future improvements

Although our stacked ensemble model outperforms its baseline models and existing methods, there remains considerable room for improvement. (i) PFGPred was trained using fusions validated from WGS data as ground truth due to the lack of experimentally confirmed fusion events in plants. As research in plant fusion biology progresses and more experimentally validated events become available, we plan to incorporate these into our datasets to develop a more reliable and biologically robust model. (ii) Currently, the model is trained on data from four plant species. In future work, we aim to expand the training dataset to include additional plant species, enabling the development of a generic and broadly applicable model for fusion gene prediction in plants. (iii) We also plan to integrate the pipeline's output into PFusionDB, improving its accessibility and usability for the research community by creating a centralized resource for plant fusion transcripts and their functional annotations. (iv) So far, our pipeline incorporates WGS and RNA-Seq data to identify fusion transcripts derived from fusion genes. In the future, we plan to integrate proteomic-level validation of fusion peptides, which will provide functional insights and strengthen the biological relevance of predicted fusion transcripts. (v) Cross-species validation demonstrated decent performance of our pipeline on human datasets. In future work, we plan to integrate non-plant datasets into model training to create a universal framework capable of accurately predicting fusion genes across both plant and non-plant species, thereby extending its utility for researchers in diverse domains.

## Supplementary Material

dsag005_Supplementary_Data

## Data Availability

PFGPred is freely available at http://www.nipgr.ac.in/PFGPred as a user-friendly web server. All the datasets and code used in this study can be found on GitHub at https://github.com/skbinfo/PFGPred.
