# Supplementary material for: PFGPred: a stack ensemble classifier for the identification of fusion genes in plants
Source: DNA Res. 2026 Jun 9;33(3):dsag005. doi: 10.1093/dnares/dsag005 (PMC13289814; doi:10.1093/dnares/dsag005)
Supplement: dsag005_Supplementary_Data [file dsag005_supplementary_data.zip › supporting_information.docx]

**PFGPred: A stack ensemble classifier for the identification of fusion genes in plants**

**Fiza Hamid^1,#^, Kanka Mukherjee^1,#^, Sakshi Chaudhary^1^, and Love Kaushik^1^ and Shailesh Kumar^1,*^**

^1^Bioinformatics Lab, BRIC-National Institute of Plant Genome Research, Aruna Asaf Ali Marg, New Delhi 110067, India.

^#^Equal contribution

^*^**Corresponding author**

Shailesh Kumar, BRIC-National Institute of Plant Genome Research (NIPGR), Aruna Asaf Ali Marg, New Delhi 110067, India. Tel:  +91-11-26735217, Fax:  +91-11-26741658

Email: [shailesh@nipgr.ac.in](mailto:shailesh@nipgr.ac.in)

The following Supporting Information is available for this article:

**Method S1. Data preprocessing before model training**

To prevent the model from artificially inflating performance by over-representing highly expressed fusions, strict deduplication was performed before model training. All fusion events were aggregated, and duplicate events characterized by identical 5' and 3' genomic breakpoint coordinates were collapsed into unique, single events. Furthermore, the validated fusion gene pairs were rigorously filtered to eliminate likely false positives arising from paralogous genes and overlapping genomic regions. Firstly, the positive and negative datasets were assigned values of 1 and 0, respectively. For categorical features, one-hot and label encoding were applied, and missing values were represented as ‘NF’ followed by binary encoding. For numerical features, imputation of missing entries using the median value was done. For the Splice site feature, each pattern was mapped to an integer, and the encodings were saved for consistent use in future datasets.

During the preprocessing stage, all direct identifiers and specific genomic locators were explicitly dropped from the feature matrix before it was fed to the algorithms. These removed features included: fusion_pair, 5_geneid, 3_geneid, CDS_LEFT_ID, CDS_RIGHT_ID, Chromosome1, Chromosome2. By stripping the data of these identifiers, the model was forced to make predictions based purely on junction characteristics, structural features, and expression metrics, eliminating the risk of gene-pair memorization.

**Method S2. Calculation of the feature importance score**

To quantify the intrinsic predictive value of each biological feature, importance scores were calculated using the Mutual Information (MI) criterion, representing the Information Gain (IG). This metric measures the statistical dependence between a feature X and the fusion label Y, capturing both linear and non-linear signals. The Information Gain, I(X; Y), was defined as:


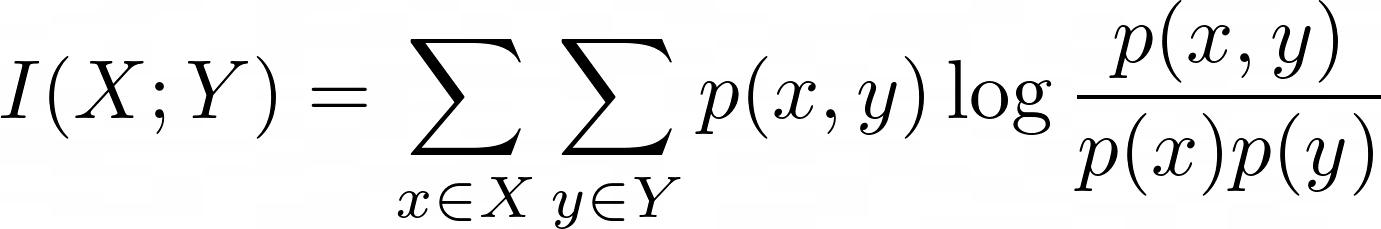


*I(X; Y): Mutual Information.*

*X,Y: The Feature and the Target (Fusion/Non-fusion).*

*p(x, y): Joint Probability.*

*p(x), p(y): Marginal Probabilities (The expected occurrence if the two were completely random).*

In this framework, p(x, y) denotes the joint probability distribution of the feature and the label, while p(x) and p(y) represent their marginal distributions. This calculation effectively measures the reduction in entropy regarding the fusion status after accounting for a specific feature.

**Method S3.** **The detailed description of the ensemble model.**

In this work, an ensemble model consisting of XGBoost, Random Forest, and LSTM as base models was used as the classification model to capture a wide variety of decision boundaries. Tree-based machine learning algorithms like XGBoost and Random Forest consistently outperform non-tree-based algorithms on tabular classification tasks. This comes from their ability to map highly non-linear relationships and seamlessly accommodate mixed categorical and numerical data, both of which are defining characteristics of our feature set. To complement the tree-based models, LSTM was included as a nonlinear gated learner within the stacking framework to capture complex interactions among heterogeneous transcriptomic and structural features and to contribute architectural diversity among the base learners.

Further, to determine the hyperparameter settings, we performed empirical tuning by evaluating a range of values for each base model and the meta-learner. Because genomic and transcriptomic datasets are inherently noisy and susceptible to overfitting, the tuning process was not aimed solely at maximizing training accuracy, but instead prioritized cross-validation stability and robust generalization to unseen data. The final selected parameters, therefore, represent the best empirical balance between model complexity and stringent regularization for our specific dataset.

**XGBoost:**

For a feature vector *xi*, XGBoost predicts a probability *ŷi* via:


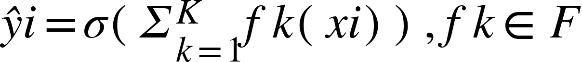


where *σ* denotes the sigmoid function, *k* is the number of trees, and *F* is the space of regression trees. Hyperparameters used include learning rate (η=0.01), maximum tree depth 6, and subsampling rate 0.8.

**Random Forest:**

The Random Forest model is trained as an ensemble of decision trees for binary classification. For a given feature vector *x*, the random forest predicts the final class *ŷ* by aggregating the votes from all individual decision trees in the forest. The predicted class (*ŷ*) is determined as the one receiving the majority of votes:


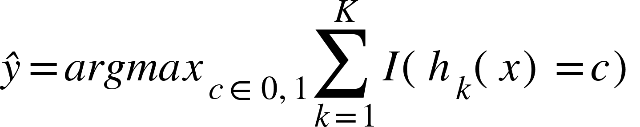


where *k* is the total number of trees, *h_k_*(*x*) is the class prediction for the kth decision tree, c represents a class label, and I is the indicator function. Each tree *h_k_* is trained on a different bootstrap sample of the original data. Hyperparameters include the number of trees (*k*=100), a maximum tree depth of 6, a minimum of 2 samples required to split a node, and a minimum of 1 sample per leaf node.

**LSTM:**

The LSTM network processes standardized feature vectors reshaped to (N,1,F), where N is the number of samples and F is the number of features. It is a single LSTM layer with 64 units and two dropout layers (p=0.4) to reduce overfitting. Hyperparameters include dense hidden layers with 16 ReLU-activated neurons and L2 regularization (λ=0.01). Predicted probabilities (*ŷi*) are produced by a final sigmoid output layer:


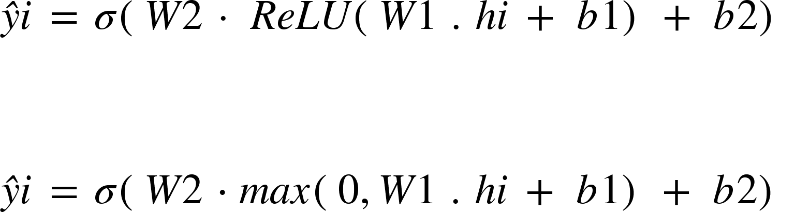


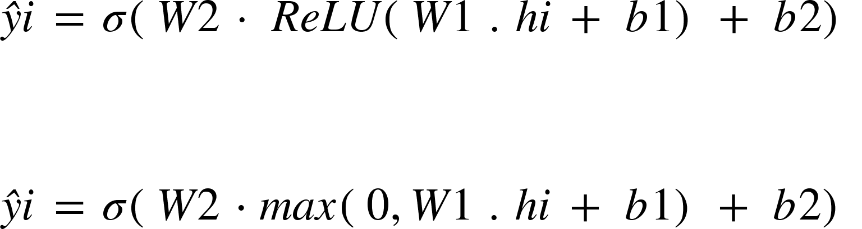


where (*h_i_*) is the LSTM output for sample *i*, and *W1*, *W2*, *b1*, *b2* are learnable parameters.

Under the present input configuration (N, 1, F), the LSTM does not utilize temporal or sequential dependencies in the conventional recurrent neural network sense, but instead behaves as a gated nonlinear transformation.

Further, the role of the LSTM was examined within the ensemble by comparing prediction correlations among base learners on the independent test set. Predictions from the LSTM were less correlated with XGBoost (r = 0.6315) and Random Forest (r = 0.6879) than the correlation observed between the two tree-based models (r = 0.8883). This suggests that the LSTM contributes complementary decision patterns, which can be beneficial in a stacked ensemble.

**Meta Model:**

We employed stacked generalization to integrate base model predictions. During training, out-of-fold predictions from XGBoost, LSTM, and Random Forest served as features for the meta learner. For each sample *i*:

*z_i_* = [*ŷi*^XGB^, *ŷi*^LSTM^, *ŷi*^RF^]

A logistic regression model is trained on *z_i_* to produce the final ensemble probability(*ŷi*):


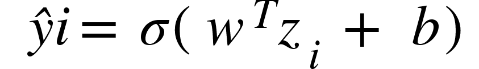


where *w* is the learnable weights and *b* is the bias term.

Our framework combines the strengths of XGBoost, Random Forest, and LSTM, capturing diverse patterns in the data and mitigating the weaknesses of individual models, while the integration of weighted predictions based on model performance further enhances the PFGPred’s reliability for fusion gene detection.

**Method S4. Performance evaluation**

To evaluate the performance of PFGPred, several metrics were considered, such as accuracy, sensitivity, specificity, MCC, and AUC, which are defined as follows:


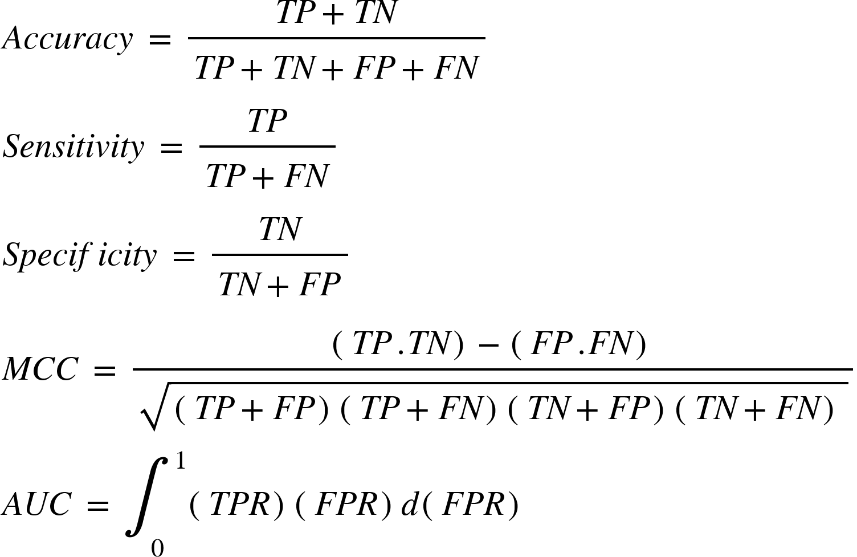


where TP, TN, FP, and FN represent the number of true positives, true negatives, false positives, and false negatives, respectively. The metrics were generated for both the training and independent datasets, and the performance between the stacked ensemble and individual base models was evaluated to determine whether PFGPred provides superior predictive capability and improved robustness over base learners.

**Supplementary Figures**

**
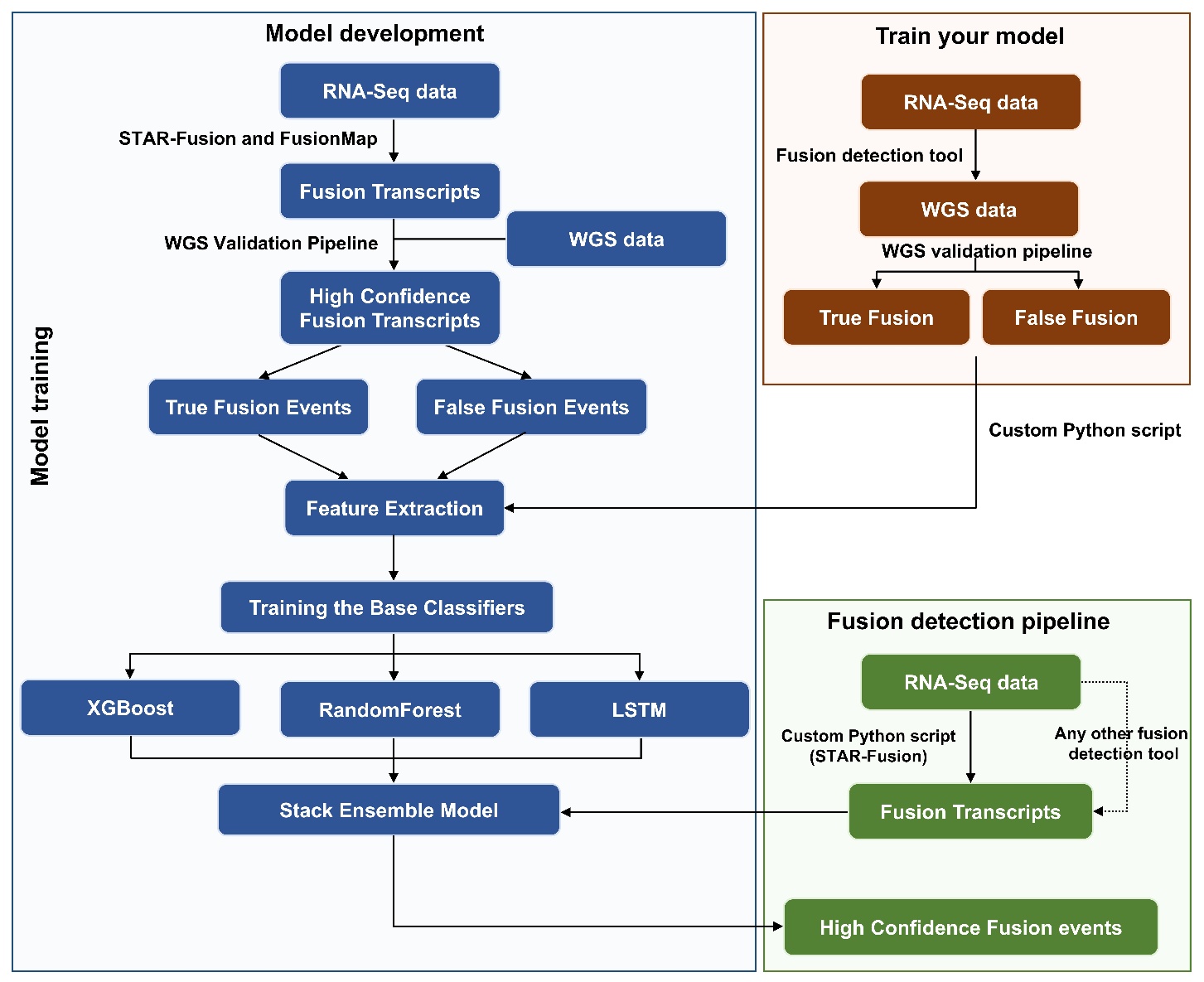
**

**Figure S1:** A schematic outline of the workflow used for detecting fusion genes.

**
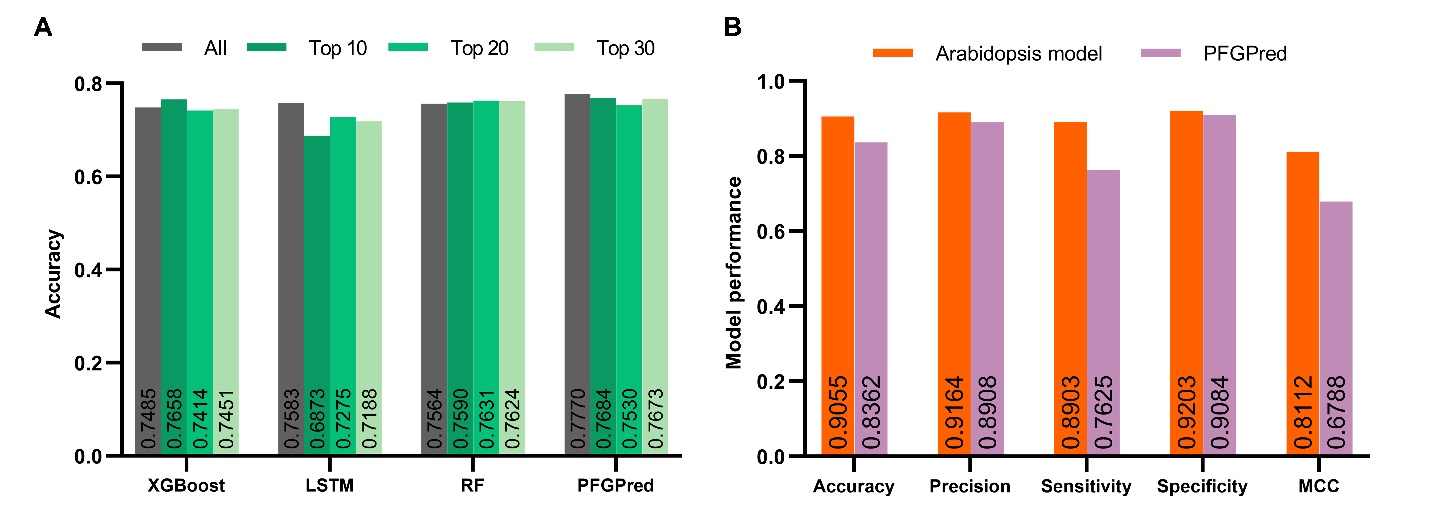
**

**Figure S2:** Performance evaluation of the model. (A) Performance evaluation of PFGPred with different sets of fusion-related features. (B) Performance comparison of the *Arabidopsis*-specific ensemble model with PFGPred on an independent dataset in terms of AUC, accuracy, precision, sensitivity, specificity, and MCC.

**
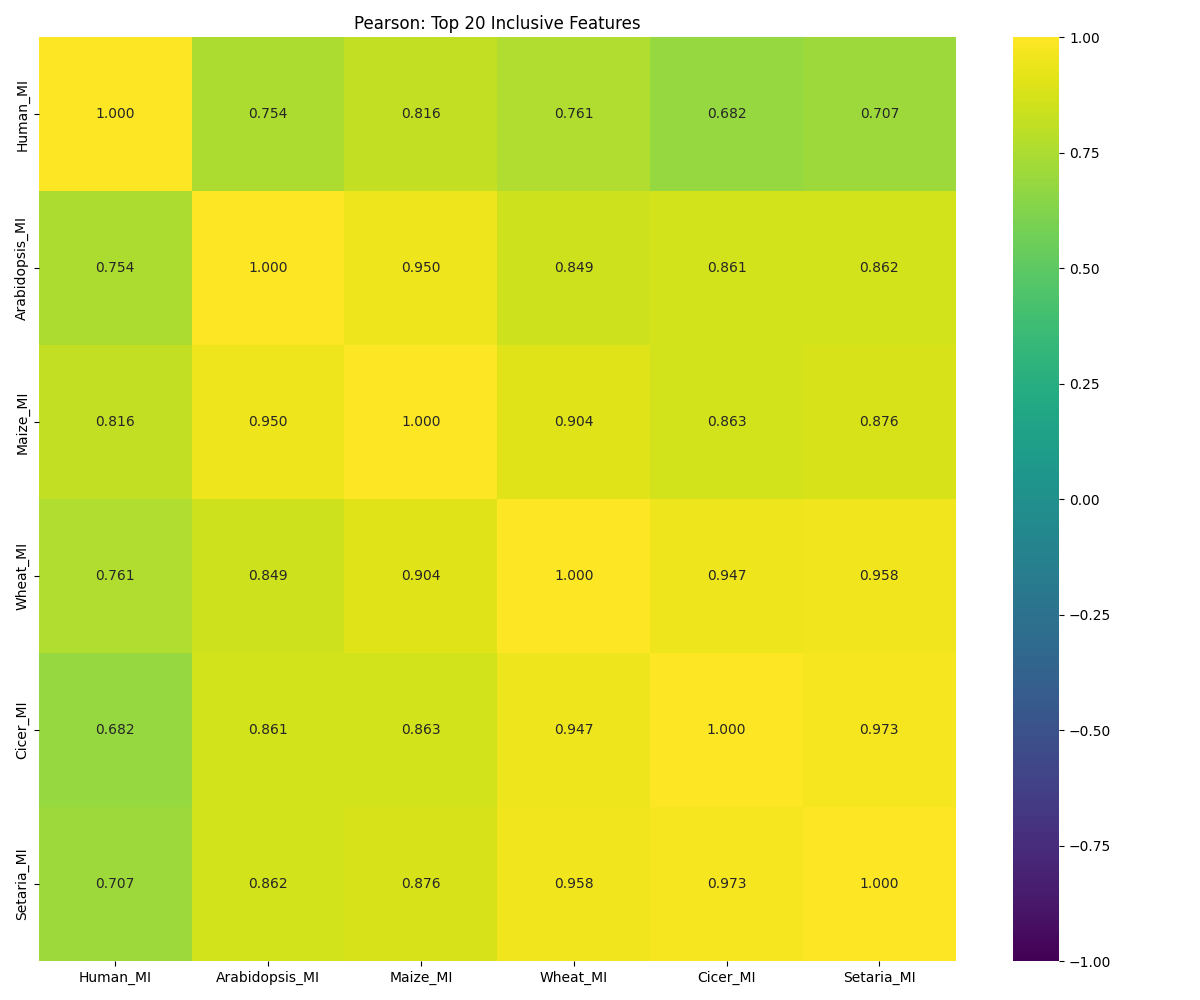
**

**Figure S3:** Pearson correlations of mutual information score for the top 20 informative features across plant species and the human dataset.

**
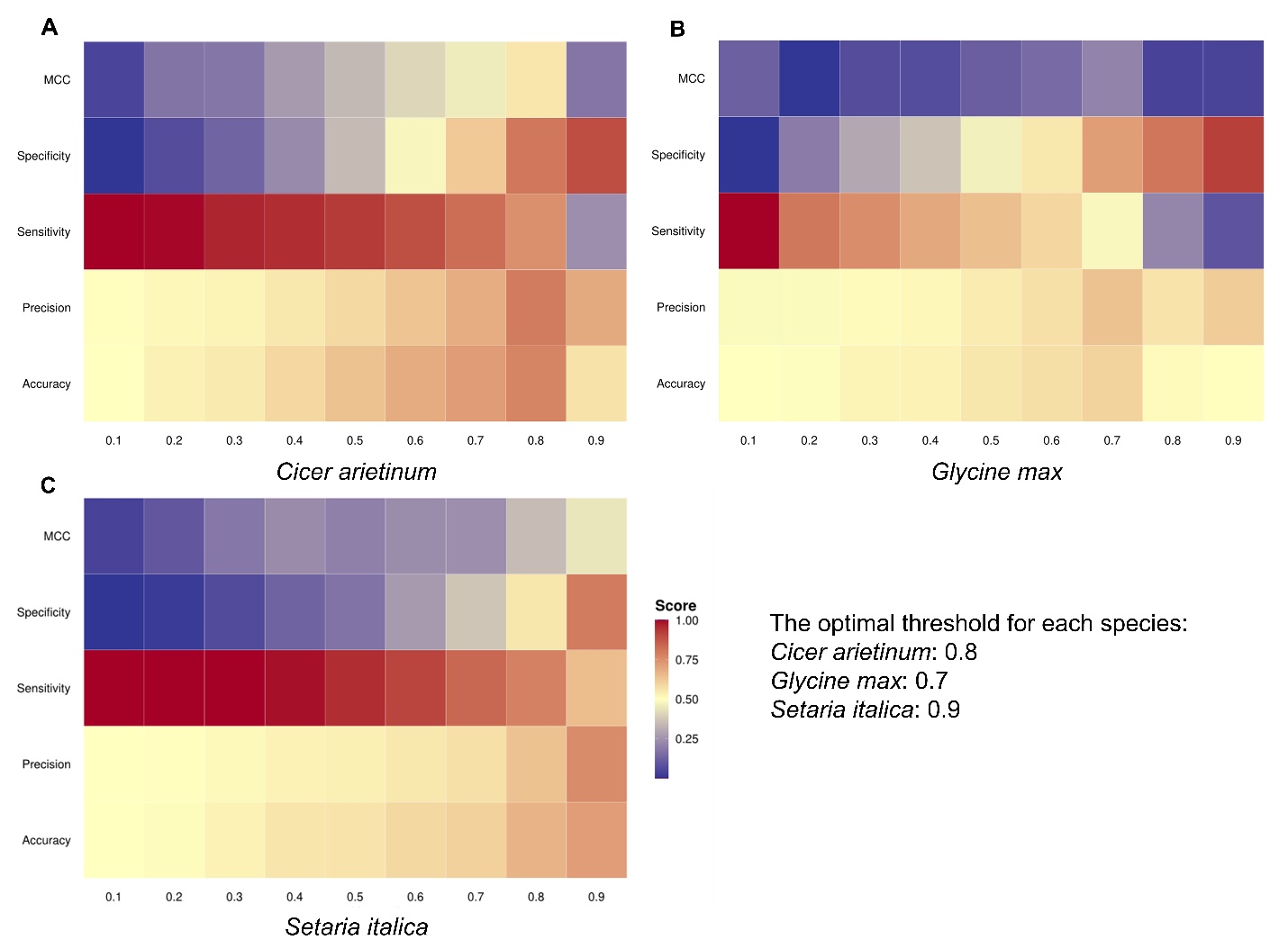
**

**Figure S4:** Performance of PFGPred on the independent test sets from multiple species at different probability thresholds. (A) Performance of the model on *Cicer arietinum*. (B) Performance of the model on *Glycine max*. (C) Performance of the model on *Setaria italica.*

**
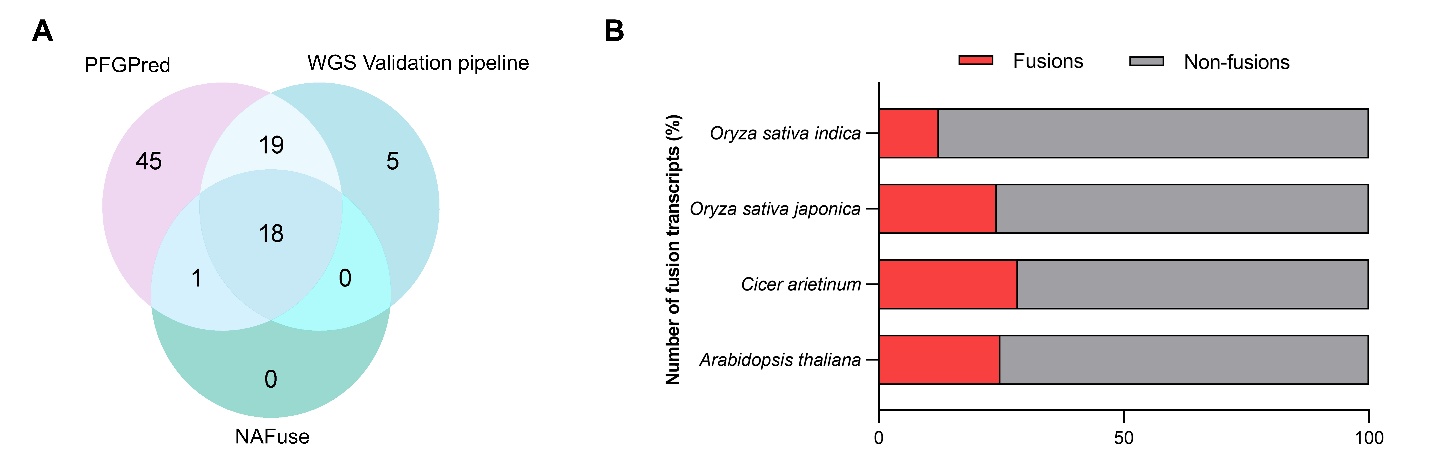
**

**Figure S5:** Comparison of PFGPred with existing fusion detection methods. (A) Overlap between fusion genes detected by PFGPred using RNA-Seq data and those identified by integrative RNA-Seq and WGS approaches, including NAFuse and the WGS fusion pipeline. (B) Number of fusion genes detected by PFGPred in the PFusionDB fusion transcript dataset.

**Supplementary Tables**

**Table S1:** List of RNA-Seq and WGS samples used for fusion detection across multiple plants. (Attached as Excel)

**Table S2** Fusion-related features used in model construction, along with their importance score.

| **Features** | **Importance score** |
| --- | --- |
| LeftBreakpoint | 0.574376064 |
| RightBreakpoint | 0.570176156 |
| 5_gene_start | 0.563879198 |
| 5_gene_end | 0.563180205 |
| 3_gene_end | 0.553937763 |
| 3_gene_start | 0.55092662 |
| 5_gene_length | 0.547646341 |
| 3_gene_length | 0.529375152 |
| Splice_Site | 0.288659087 |
| Splice_Pattern_Class_NonCanonicalPattern | 0.136389702 |
| Splice_Pattern_Class_CanonicalPattern | 0.134359396 |
| 5_loc_M | 0.130185893 |
| Total_Mapped_Reads | 0.11586394 |
| 3_loc_M | 0.113812817 |
| alternate_junction_count | 0.080555709 |
| FFPM | 0.080198942 |
| exon_count3 | 0.076854821 |
| exon_count5 | 0.064709197 |
| Right_Exon | 0.057780691 |
| 5_loc_S | 0.051937137 |
| Left_Exon | 0.049705411 |
| 5_loc_E | 0.045418134 |
| 3_loc_E | 0.045145199 |
| Splice_Pattern_Unknown | 0.043317114 |
| Total_Count_(SC+RC) | 0.03706791 |
| 3_loc_S | 0.035389709 |
| Chromosome_Feature_Interchromosomal | 0.035354261 |
| Splice_Pattern_InFrame | 0.034846689 |
| Chromosome_Feature_Intrachromosomal | 0.028430899 |
| alternative_junction_Yes | 0.018376873 |
| alternative_junction_No | 0.016325041 |
| Reciprocal_Fusion_Yes | 0.008736902 |
| LeftStrand__ | 0.006006019 |
| LeftStrand_+ | 0.005897705 |
| RightStrand__ | 0.003832937 |
| Splice_Pattern_FrameShift | 0.003689691 |
| 3_loc_O | 0.002889405 |
| Reciprocal_Fusion_No | 0.001626722 |
| 5_loc_O | 0.00060805 |
| RightStrand_+ | 0.000501169 |
| Same_Strand_Yes | 0.000342178 |

**Table** **S3** Performance comparison of different baseline models and PFGPred.

| **Model** | **Accuracy** | **Precision** | **Sensitivity** | **Specificity** | **F1_Score** | **MCC** | **AUC** | **Log_Loss** | **Positive_Count** | **Negative_Count** |
| --- | --- | --- | --- | --- | --- | --- | --- | --- | --- | --- |
| PFGPred | 0.95949 | 0.942044 | 0.979223 | 0.939757 | 0.960274 | 0.919697 | 0.991361 | 0.117733 | 11022 | 11022 |
| LightGBM_Train | 0.905734 | 0.882615 | 0.935946 | 0.875522 | 0.908498 | 0.812953 | 0.954779 | 0.377192 | 11022 | 11022 |
| XGBoost_Train | 0.90519 | 0.880798 | 0.937216 | 0.873163 | 0.908132 | 0.812047 | 0.954167 | 0.377082 | 11022 | 11022 |
| RandomForest_Train | 0.895164 | 0.88432 | 0.909272 | 0.881056 | 0.896623 | 0.790643 | 0.962949 | 0.286194 | 11022 | 11022 |
| LSTM_Train | 0.959762 | 0.938935 | 0.983488 | 0.936037 | 0.960695 | 0.920562 | 0.992299 | 0.105369 | 11022 | 11022 |
| SVM_Train | 0.93327 | 0.923924 | 0.944293 | 0.922246 | 0.933997 | 0.86675 | 0.974454 | 0.196834 | 11022 | 11022 |
| KNN_Train | 0.956042 | 0.931867 | 0.984032 | 0.928053 | 0.957239 | 0.913517 | 0.995301 | 0.082279 | 11022 | 11022 |
| LASSO_Train | 0.812421 | 0.805302 | 0.824079 | 0.800762 | 0.814582 | 0.625011 | 0.887836 | 0.424809 | 11022 | 11022 |
| PFGPred | 0.777027027 | 0.795673077 | 0.745495495 | 0.808558559 | 0.769767442 | 0.555159074 | 0.847366511 | 0.549923123 | 1332 | 1332 |
| LightGBM_Test | 0.726351 | 0.747334 | 0.683934 | 0.768769 | 0.71423 | 0.454341 | 0.822974 | 0.53906 | 1332 | 1332 |
| XGBoost_Test | 0.748498498 | 0.761041009 | 0.724474474 | 0.772522523 | 0.742307692 | 0.49757168 | 0.827960562 | 0.527747425 | 1332 | 1332 |
| LSTM_Test | 0.758258258 | 0.774760383 | 0.728228228 | 0.788288288 | 0.750773994 | 0.517450636 | 0.837517134 | 0.570266279 | 1332 | 1332 |
| RandomForest_Test | 0.756381381 | 0.758516276 | 0.752252252 | 0.760510511 | 0.755371278 | 0.512780249 | 0.821220996 | 0.519684667 | 1332 | 1332 |
| SVM_Test | 0.77515 | 0.85826 | 0.659159 | 0.891141 | 0.745648 | 0.565733 | 0.851639 | 0.611488 | 1332 | 1332 |
| KNN_Test | 0.769144 | 0.841104 | 0.663664 | 0.874625 | 0.741922 | 0.550682 | 0.808435 | 5.276514 | 1332 | 1332 |
| LASSO_Test | 0.713964 | 0.721617 | 0.696697 | 0.731231 | 0.708938 | 0.428183 | 0.770165 | 0.631231 | 1332 | 1332 |

**Table S4** List of fusion genes validated through long-read sequencing. (Attached as Excel).

**Table S5** Fusion genes coding potential predicted through RNASamba. (Attached as Excel).

**Table S6** Performance of the PFGPred in terms of precision, sensitivity, specificity, MCC, and accuracy using different sets of features.

| **Features** | **Model** | **Precision** | **Sensitivity** | **Specificity** | **MCC** | **AUC** |
| --- | --- | --- | --- | --- | --- | --- |
| **Top 10** | **XGBoost** | 0.742465753 | 0.813813814 | 0.717717718 | 0.53400286 | 0.7798412 |
|  | **LSTM** | 0.639619474 | 0.858108108 | 0.516516517 | 0.39860108 | 0.7230741 |
|  | **RandomForest** | 0.746428571 | 0.784534535 | 0.733483483 | 0.51869437 | 0.7912079 |
|  | **PFGPred** | 0.765799257 | 0.773273273 | 0.763513514 | 0.53681235 | 0.7883658 |
| **Top 20** | **XGBoost** | 0.76202119 | 0.701951952 | 0.780780781 | 0.48423961 | 0.8226873 |
|  | **LSTM** | 0.71988389 | 0.744744745 | 0.71021021 | 0.4552265 | 0.7936743 |
|  | **RandomForest** | 0.760594796 | 0.768018018 | 0.758258258 | 0.52630134 | 0.8255223 |
|  | **PFGPred** | 0.768740032 | 0.723723724 | 0.782282282 | 0.50687582 | 0.8233656 |
| **Top 30** | **XGBoost** | 0.763945028 | 0.709459459 | 0.780780781 | 0.49149188 | 0.8257461 |
|  | **LSTM** | 0.755030621 | 0.647897898 | 0.78978979 | 0.44216141 | 0.833047 |
|  | **RandomForest** | 0.76988417 | 0.748498498 | 0.776276276 | 0.52497735 | 0.8353734 |
|  | **PFGPred** | 0.796173045 | 0.718468468 | 0.816066066 | 0.53709867 | 0.848372 |
| **All features** | **XGBoost** | 0.761041009 | 0.724474474 | 0.772522523 | 0.49757168 | 0.8279606 |
|  | **LSTM** | 0.774760383 | 0.728228228 | 0.788288288 | 0.51745064 | 0.8375171 |
|  | **RandomForest** | 0.758516276 | 0.752252252 | 0.760510511 | 0.51278025 | 0.821221 |
|  | **PFGPred** | 0.795673077 | 0.745495495 | 0.808558559 | 0.55515907 | 0.8473665 |

**Table S7** List of fusion genes predicted by PFGPred from PFusionDB, along with their probability scores. (Attached as Excel)

**Table S8** List of experimentally validated fusions detected by PFGPred.

| **Fusion gene** | **Organism** | **Validation method** | **PFGPred Score** | **Reference** |
| --- | --- | --- | --- | --- |
| AT1G79040--AT1G67090 | *Arabidopsis thaliana* | LC-MS (Protein) | 0.9390140445 | [36] |
| BGIOSGA017770--BGIOSGA004569 | *Oryza sativa indica* | qRT-PCR | 0.9249372171 |  |
| BGIOSGA028186--BGIOSGA014361 | *Oryza sativa indica* | LC-MS (Protein) | 0.9470883007 |  |
| BGIOSGA029125--BGIOSGA031172 | *Oryza sativa indica* | LC-MS (Protein) | 0.8351359271 |  |
| BGIOSGA013731--BGIOSGA010198 | *Oryza sativa indica* | LC-MS (Protein) | 0.9609660262 |  |
| Os08g0534200--Os02g0305800 | *Oryza sativa japonica* | qRT-PCR | 0.903214579 |  |
| LOC101489433--LOC101489100 | *Cicer arietinum* | qRT-PCR | 0.9622477029 | [12] |
| LOC101515613--LOC113787786 | *Cicer arietinum* | qRT-PCR | 0.931817119 |  |
| LOC101494819--LOC101493433 | *Cicer arietinum* | qRT-PCR | 0.9004783373 |  |
